# SSRI prescription during acute COVID-19 and risk of Long COVID symptoms and conditions among patients with depression

**DOI:** 10.64898/2026.07.06.26357401

**Published:** 2026-07-09

**Authors:** Zachary Butzin-Dozier, Yunwen Ji, Lin-Chiun Wang, Manav Kumar, A. Jerrod Anzalone, Eric Hurwitz, Ariana Budhihartanto, Rena C. Patel, Alan Hubbard, Jodi Halpern

**Affiliations:** 1 Stanford University School of Medicine, Stanford, CA, USA; 2 School of Public Health, University of California, Berkeley, Berkeley, CA USA; 3 University of Nebraska Medical Center, Omaha, NE, USA; 4 University of North Carolina at Chapel Hill, Chapel Hill, NC, USA; 5 University of Alabama at Birmingham, Birmingham, AL, USA

## Abstract

**Background:**

Long COVID is a syndrome characterized by symptoms and conditions across all biological systems. This breadth of Long COVID phenotypes impedes efforts to identify the mechanistic pathways of Long COVID. Low serotonin may play a role in long-term sequelae of COVID-19, and selective serotonin reuptake inhibitors (SSRIs) may prevent these sequelae. Evaluation of the relationship between SSRIs and distinct categories of symptoms and conditions associated with Long COVID can highlight the mechanistic pathways that drive these relationships.

**Methods:**

We evaluated electronic health record data from a retrospective cohort of patients in the National Clinical Cohort Collaborative with comorbid depression and COVID-19 between October 2021 and February 2024. We estimated the relationship between SSRI prescription (versus no SSRI prescription) during acute COVID-19 and the one-year cumulative incidence of Long COVID-related conditions and symptoms across 14 human phenotype ontology categories. We applied Super Learner and targeted maximum likelihood estimation to estimate risk ratios while adjusting for confounders of interest and correcting for false discoveries from repeated testing.

**Results:**

We evaluated EHR data from 542,938 patients. We found that patients who were prescribed SSRIs during COVID-19 had a significantly lower risk of symptoms and conditions related to gastrointestinal factors (adjusted risk ratio (aRR) 0.95, 95% CI 0.92, 0.97), general health (aRR 0.91, 95% CI 0.88, 0.95), headaches (aRR 0.96, 95% CI 0.92, 0.99) and skin (aRR 0.92, 95% CI 0.87, 0.98).

**Discussion:**

We found that the prescription of SSRIs during acute COVID-19 was associated with a significantly lower risk of post-COVID sequelae related to gastrointestinal, headache-related, skin-related, and general symptoms and conditions, compared with no SSRI prescription. These findings highlight the role of serotonin in Long COVID and specific sequelae that may be reduced by SSRIs.

## INTRODUCTION

Patients infected with SARS-CoV-2 experience a wide range of symptoms that typically subside within several weeks, but a subgroup of patients experiences long-term symptoms after the acute infection has resolved. Given the global prevalence of COVID-19, now that SARS-CoV-2 has become endemic, it is crucial to understand the pathways that drive long-term consequences of infection and how to interrupt these outcomes.

A recent study found that low serotonin may be a key driver of many post-COVID-19 symptoms.^1^ This theory highlights the biological mechanism where (1) SARS-CoV-2 infection elicits the release of virus-induced interferons, (2) these interferons prevent the uptake of tryptophan, which is a precursor to serotonin (i.e., interferons prevent the production of serotonin), (3) low serotonin impairs vagal functioning, neurologic functioning, and coagulation.^1,2^

Observational studies have found that use of SSRIs during acute COVID-19 is associated with a lower subsequent risk of Long COVID.^1–3^ On the other hand, the COVID-OUT randomized trial did not find a significant relationship between COVID-19 outpatient assignment to an SSRI and subsequent risk of Long COVID.^4^ Given the breadth of post-COVID phenotypes and the multiple hypothesized biological mechanisms that drive them, considerable effort has been devoted to identifying unique clusters of post-COVID phenotypes.^5,6^ Understanding the relationships between serotonin and unique post-COVID symptoms may provide important information on the clusters of symptoms that may be treated with SSRIs and the clusters of patients who have Long COVID driven by low serotonin.

In this study, we sought to evaluate the relationship between SSRI prescription during acute COVID-19 and the subsequent risk of post-COVID symptoms among a sample of electronic health records from patients with depression in the National Clinical Cohort Collaborative (N3C).

## METHODS

### Sample:

We analyzed data from electronic health records (EHR) from patients in the National Clinical Cohort Collaborative (N3C), which is a large, national open source of health data that contains information for more than 21 million patients.^7,8^ Our sample included patients diagnosed with acute COVID-19 between October 1, 2021 and February 1, 2024 with comorbid depression. N3C defines COVID-19 positivity as (A) at least one laboratory diagnostic positive result or (B) a provider diagnosis (ICD-10-CM U07.1). We defined the index date for COVID-19 as the earliest of the two events.^9^

### Exposure:

Our exposure of interest was prescription of selective serotonin reuptake inhibitors (SSRI) during acute COVID-19. To be defined as an SSRI recipient, the patient must have been prescribed an SSRI at least 30 days before acute COVID-19 and did the prescription did not stop before COVID-19. SSRIs include fluoxetine, sertraline, paroxetine, citalopram, escitalopram, fluvoxamine, and vilazodone.

### Outcomes:

Our outcomes of interest were 211 conditions and symptoms associated with Long COVID (hereafter referred to as “symptoms”) between 1 and 12 months after acute COVID-19. We selected this time period at-risk in accordance with Centers for Disease Control and Prevention guidelines.^10^ These 211 symptoms were drawn from previous studies that have identified and validated their use as metrics of Long COVID (post-acute sequelae of COVID-19)^11^ from the Human Phenotype Ontology (HPO) (for full list of Long COVID symptoms and conditions, see Supplemental Material 1).^6,12–14^ We summarized these symptoms across 14 symptom categories (where each category is a binary indicator of whether a patient had any symptom in that category) and as a single binary indicator (whether the patient had any post-COVID symptom). We excluded 6 symptom categories (and 76 symptoms) associated with depression and SSRI use, as they would be more frequently evaluated in patients with depression who were prescribed SSRIs compared to patients not prescribed SSRIs (i.e., measurement bias). We excluded neuropsychiatric symptom categories related to emotion and mood, behavioral, sleep, cognitive dysfunction, memory, and findings.^15^

### Covariates:

Our analysis adjusted for the following confounders at baseline (acute COVID-19): healthcare utilization rate (healthcare interactions per month before SARS-CoV-2 infection), sex, age at acute SARS-CoV-2 infection, race/ethnicity, region of residence, body mass index (BMI), tobacco smoking status, obesity, diabetes, chronic lung disease, heart failure, hypertension, use of systemic corticosteroids, depression severity, anxiety, antipsychotic medication use, benzodiazepine medication use, whether the patient was immunocompromised, the number of COVID-19 vaccination doses before infection, date of COVID-19, and Charlson Comorbidity Index.^16^ We adjusted for the following county-level socioeconomic variables: the percentage of the county below the poverty line and the county’s social deprivation index score. We considered monitoring as a source of informative right censoring, where we considered a patient as informatively censored if they had fewer than 2 healthcare interactions or died within the 12 months following acute COVID-19, and evaluated our causal parameter under a scenario of universal monitoring (i.e., no censoring) consistent with previous studie.^2,17–19^

### Analysis:

We applied Super Learner and Targeted Maximum Likelihood Estimation, which are effective for this analytic setting that relies on electronic health records with a high proportion of missingness.^18–21^ We used Super Learner, an ensemble machine learning algorithm, to maximize the prediction of the outcome, post-COVID symptoms, as well as the treatment and censoring mechanisms.^17,22,23^ We included the learners: generalized linear models (“SL.glm”), GLM net (“SL.glmnet”), and XGBoost (“SL.xgboost”).^23^ Next, we applied targeted maximum likelihood estimation to estimate the risk ratio comparing the risk of a given symptom category in patients prescribed an SSRI during COVID-19 to that in patients not prescribed an SSRI during COVID-19.^18–21^ Targeted maximum likelihood estimation is a doubly-robust estimator, meaning that it provides valid estimates as long as either the outcome regression or treatment mechanism is estimated consistently.^18–21^ During the one-year follow-up period, we considered mortality and lack of healthcare utilization as sources of informative censoring.^17^ We corrected our estimates for multiple testing using the Benjamini-Hochberg correction.

## RESULTS

We analyzed EHR data from 542,938 patients with comorbid depression and COVID-19; 134,595 (74% female) were prescribed SSRIs, and 408,343 (71% female) were not prescribed SSRIs during acute COVID-19. SSRI patients had an average Charlson Comorbidity Index Score of 2.36, while non-SSRI patients had an average of 1.96. Approximately 9% of SSRI patients had severe depression, while 8% of non-SSRI patients had severe depression. Patients prescribed SSRIs had, on average, 1.8 healthcare interactions per month, while those not prescribed SSRIs had 1.48 per month.

**Figure 1.**
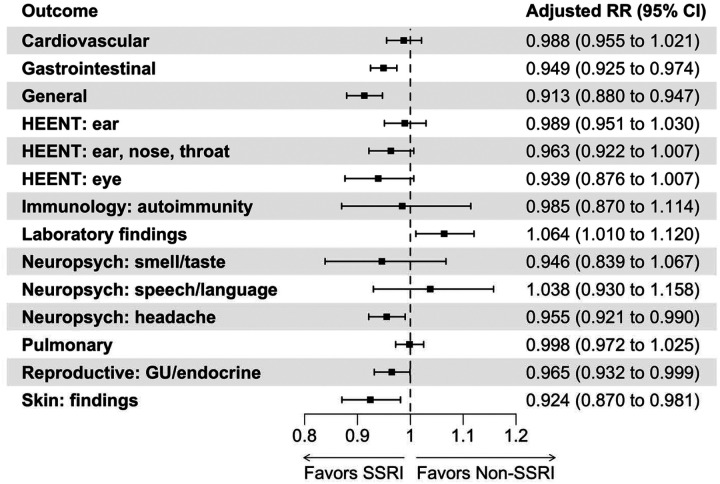
Adjusted relationships between SSRI prescription (*n* = 134,595) during acute COVID-19 and subsequent 12-month cumulative incidence of conditions and symptoms associated with Long COVID, compared to no SSRI prescription (*n* = 408,343), among patients with depression. SSRI: Selective serotonin reuptake inhibitor, RR: Risk ratio, CI: Confidence interval, HEENT: Head, eyes, ears, nose, and throat, Neuropsych: Neuropsychological, GU: genitourinary

We found that patients who were prescribed SSRIs during COVID-19 had a significantly lower risk of gastrointestinal (adjusted risk ratio (aRR) 0.95, 95% CI 0.92, 0.97), general (aRR 0.91, 95% CI 0.88, 0.95), headache (aRR 0.96, 95% CI 0.92, 0.99) reproductive (aRR 0.96, 95% CI 0.93, 1.00), and skin-related (aRR 0.92, 95% CI 0.87, 0.98) symptoms and conditions (Figure 1, Table 2). Reproductive symptoms and conditions did not retain significance after FDR correction. We did not detect a significant relationship between SSRI prescription and cardiovascular (aRR 0.99, 95% CI 0.96, 1.02), ear-related (aRR 0.99, 95% CI 0.95, 1.03), ear-nose-throat-related (aRR 0.96, 95% CI 0.92, 1.01), eye-related (aRR 0.94, 95% CI 0.88, 1.01), autoimmunity-related (aRR 0.98, 95% CI 0.87, 1.11), smell and taste related (aRR 0.95, 95% CI 0.84, 1.07), speech and language-related (aRR 1.04, 95% CI 0.93, 1.16), or pulmonary (aRR 1.00, 95% CI 0.97, 1.03) symptoms and conditions. Patients prescribed SSRIs during acute COVID-19 had a significantly higher risk of abnormal laboratory findings (aRR 1.06, 95% CI 1.01, 1.12), compared to patients not prescribed an SSRI, but this observation did not retain significance after FDR correction.

## DISCUSSION

We found that patients who were prescribed SSRIs during acute COVID-19 had a significantly lower risk of subsequent gastrointestinal, headache-related, skin-related, and general symptoms and conditions, compared to patients not prescribed SSRIs, among patients with depression and comorbid COVID-19 between October 1, 2021, and February 1, 2024. These findings are consistent with previous observational studies that have found that the use of SSRIs during acute COVID-19 was associated with a lower risk of Long COVID,^1–3^ although these findings have not been replicated in a randomized context.^4^

These findings support hypotheses about the importance of serotonin, which has key receptors in both the brain and the gut, across several Long COVID phenotypes and pathways.^24^ A key hypothesized mechanism of Long COVID posits that residual SARS-CoV-2 viral particles in Long COVID patients trigger the sustained release of virus-induced Type 1 interferon, which prevents the production of tryptophan, which is a precursor to serotonin.^1,2^ This reduced production of serotonin impairs neurologic and vagal functioning, while contributing to hypercoagulation.^1,2^ *Gastrointestinal* symptoms and conditions are central to this pathway, as serotonin and tryptophan are gut-derived, and vagal functioning is a key component of gut-brain communication.^1,24,25^ Furthermore, impaired vagal functioning and hypercoagulation via insufficient peripheral serotonin may explain the increased incidence of *headaches*, *skin-related*, and *general* symptoms.^24^ Cumulatively, these finding highlights the potential role of serotonin in contributing to disparate Long COVID-related phenotypes.

### Strengths and limitations

Our categorization of patient symptom profiles is a potential limitation of this study. We chose to summarize patient symptom burden across 14 categories as binary indicators (i.e., 14 yes/no values) in order to retain phenotype specificity while maintaining study power and maximizing interpretability. Residual confounding by symptom severity or symptom duration remains plausible, although we lacked reliable data on these metrics. Furthermore, our counterfactual intervention contrasted outcomes between patients with no symptoms versus patients with one or more symptoms, which leaves the possibility of unique relationships among patients with high symptom burden (e.g., 5 or more symptoms), although our sample size of high symptom burden patients was small and therefore evaluations would be vulnerable to near positivity violations. Another limitation is our exclusion of symptoms that were related to depression or SSRI use. Including these symptoms would be vulnerable to measurement bias, as patients prescribed SSRIs would be evaluated for them more frequently. Future studies using active outcome ascertainment (i.e., not vulnerable to measurement bias) should include these symptoms in their assessments. The generalizability of N3C has been described previously as a limitation. While N3C is a broad, national sample, it overrepresents academic research centers and patients with high healthcare-seeking behaviors. Therefore, our sample has a disproportionately high socioeconomic status, is older, and has more comorbidities than the general population.^6,12,26^

The data source, N3C, was a strength of this study, as it includes high-dimensional EHR data from a large patient sample.^8^ The analysis approach, using Super Learner and targeted maximum likelihood estimation, was a second strength of this study.^18,19,22,27^ This approach allows us to flexibly model the outcome regression, treatment mechanism, and censoring mechanism with minimal parametric assumptions while adjusting for confounders of interest. This setting, where we face informative censoring and a difficult-to-characterize treatment mechanism, benefits from the robustness of this approach.

## CONCLUSIONS

We found that the prescription of SSRIs during acute COVID-19 was associated with a significantly lower risk of post-COVID sequelae related to gastrointestinal, headache-related, skin-related, and general symptoms and conditions, compared to no SSRI prescription, among patients with depression and comorbid COVID-19 between October 1, 2021, and February 1, 2024. These findings highlight the role of serotonin in Long COVID and highlight specific sequelae that may be reduced by SSRIs.

## Supplementary Material

1

## Figures and Tables

**Table 1.** Characteristics of patients with depression who were prescribed serotonin reuptake inhibitors during acute COVID-19.

Characteristic	Value	SSRIs: Count (Proportion)	No SSRIs: Count (Proportion)	Total Count (Proportion)
Total		134595 (0.25)	408343 (0.75)	542938 (1)
Sex	Female	100132 (0.74)	290954 (0.71)	391086 (0.72)
Measurements	Age: mean (SD)	51.7 (19.4)	49.77 (19.6)	50.25 (19.57)
	BMI: mean (SD)	34.17 (9.62)	33.39 (9.51)	33.59 (9.54)
Ethnicity	White Non-Hispanic	101196 (0.75)	289604 (0.71)	390800 (0.72)
	Blackor African American Non-Hispanic	15552 (0.12)	49251 (0.12)	64803 (0.12)
	Hispanic or Latino Any Race	9715 (0.07)	36083 (0.09)	45798 (0.08)
	Unknown	4024 (0.03)	14738 (0.04)	18762 (0.03)
	Other Non-Hispanic	781 (0.01)	6780 (0.02)	7561 (0.01)
	American Indian Non-Hispanic	627 (0)	2361 (0.01)	2988 (0.01)
	Asian or Pacific Islander Non-Hispanic	2700 (0.02)	9526 (0.02)	12226 (0.02)
Medical Conditions	Tobacco Smoker	33202 (0.25)	86343 (0.21)	119545 (0.22)
	Obese	84985 (0.63)	231129 (0.57)	316114 (0.58)
	Diabetes Uncomplicated	36142 (0.27)	94674 (0.23)	130816 (0.24)
	Diabetes Complicated	26063 (0.19)	67271 (0.16)	93334 (0.17)
	Chronic Lung Disease	47932 (0.36)	129312 (0.32)	177244 (0.33)
	Heart Failure	19746 (0.15)	45778 (0.11)	65524 (0.12)
	Hypertension	71805 (0.53)	193674 (0.47)	265479 (0.49)
	Anxiety	103512 (0.77)	259908 (0.64)	363420 (0.67)
	Immunocompromised	20635 (0.15)	53399 (0.13)	74034 (0.14)
	Charlson Comorbidity Index Score: mean (SD)	2.36 (2.78)	1.96 (2.51)	2.06 (2.59)
Depression	Mild Depression	17682 (0.13)	42755 (0.1)	60437 (0.11)
	Severe Depression	12490 (0.09)	31337 (0.08)	43827 (0.08)
Drugs	Systemic Corticosteroids	89295 (0.66)	207367 (0.51)	296662 (0.55)
	Antipsychotic Medication	19455 (0.14)	43149 (0.11)	62604 (0.12)
	Benzodiazepine	43944 (0.33)	94614 (0.23)	138558 (0.26)
Medical Utilization	Number of Previous Vaccinations: mean (SD)	1.19 (1.4)	1.14 (1.37)	1.15 (1.38)
	Visits per Month: mean (SD)	1.8 (1.72)	1.48 (1.66)	1.56 (1.68)
	2 Visits Within Year of COVID	127479 (0.95)	362941 (0.89)	490420 (0.9)
Socioeconomic Factors	Poverty Rate: mean (SD)	15.31 (5.19)	14.91 (5.11)	15 (5.13)
	Social Deprivation Index Score: mean (SD)	45.48 (27.15)	45.1 (28.83)	45.19 (28.44)

**Table 2.** Unadjusted relationships between SSRI prescription (*n* = 134,595) during acute COVID-19 and subsequent 12-month cumulative incidence of conditions and symptoms associated with Long COVID, compared to no SSRI prescription (*n* = 408,343) and false discovery rate corrected p-values, among patients with depression.

Outcome	Count SSRI Patients	Count Non-SSRI Patients	Number of SSRI Patients with a LC symptom	Number of non-SSRI Patients with a LC symptom	Risk SSRI Patients	Risk Non-SSRI Patients	Unadjusted RR (95% Cl)	FDR Corrected Adjusted P value
**Cardiovascular**	134595	408343	23474	55773	0.174	0.137	1.28 (1.26, 1.30)	0.65
**Gastrointestinal (**	134595	408343	39829	101047	0.296	0.247	1.20 (1.18, 1.21)	0
**General**	134595	408343	27308	72338	0.203	0.177	1.15 (1.13, 1.16)	0
**HEENT: ear**	134595	408343	14067	33761	0.105	0.083	1.26 (1.24, 1.29)	0.7
**HEENT: ear, nose, and throat**	134595	408343	8558	20725	0.064	0.051	1.25 (1.22, 1.28)	0.17
**HEENT: eye**	134595	408343	6386	16312	0.047	0.04	1.19 (1.15, 1.22)	0.15
**Immunology: autoimmunity**	134595	408343	1203	2800	0.009	0.007	1.29 (1.22, 1.39)	0.87
**Laborator y findings**	134595	408343	10887	24869	0.081	0.061	1.33 (1.30, 1.36)	0.05
**Neuropsy chiatric: smell and taste**	134595	408343	451	996	0.003	0.002	1.37 (1.23, 1.54)	0.57
**Neuropsychiatric: speech and language**	134595	408343	1295	2690	0.01	0.007	1.46 (1.37, 1.56)	0.65
**Neuropsychiatric: headache**	134595	408343	18012	45529	0.134	0.111	1.20 (1.18, 1.22)	0.04
**Pulmonary**	134595	408343	32218	79147	0.239	0.194	1.23 (1.22, 1.25)	0.91
**Reproductive: genitourinary, endocrine, metabolism**	134595	408343	18746	44994	0.139	0.11	1.26 (1.24, 1.28)	0.11
**Skin: findings**	134595	408343	7574	18740	0.056	0.047	1.23 (1.20, 1.26)	0.04

SSRI: Selective serotonin reuptake inhibitor, RR: Risk ratio, CI: Confidence interval, HEENT: Head, eyes, ears, nose, and throat, Neuropsych: Neuropsychological, GU: genitourinary, FDR: false discovery rate

## Data Availability

All analytic code is available upon request from the N3C Enclave. Access to study data may be requested in the N3C Enclave as “legacy data” pending N3C approval. Access to the N3C Data Enclave is managed by NCATS (https://ncats.nih.gov/research/research-activities/n3c/resources/data-access). Interested researchers must first complete a data use agreement and then a data use request to access the N3C Data Enclave. Once access is granted, the N3C data use committee must review and approve all data use, and the publication committee must approve all publications involving N3C data.
