# Supplementary material for: SSRI prescription during acute COVID-19 and risk of Long COVID symptoms and conditions among patients with depression
Source: medRxiv. 2026 Jul 9:2026.07.06.26357401. Preprint. [Version 1] doi: 10.64898/2026.07.06.26357401 (PMC13370595; doi:10.64898/2026.07.06.26357401)
Supplement: 1 [file NIHPP2026.07.06.26357401V1-supplement-1.pdf]

medRxiv preprint doi: https://doi.org/10.64898/2026.07.06.26357401; this version posted July 9, 2026. The copyright holder for this preprint (which was not certified by peer review) is the author/funder, who has granted medRxiv a license to display the preprint in perpetuity.
It is made available under a CC-BY 4.0 International license .

SUPPLEMENTAL MATERIALS

Supplemental Material 1. Symptoms and conditions associated with Long COVID.

HPO_id	HPO_label	category
**HP:0030784**	Anomic aphasia	neuropsychiatric-speech-language
**HP:0002381**	Aphasia	neuropsychiatric-speech-language
**HP:0033849**	Bilingual aphasia	neuropsychiatric-speech-language
**HP:0002427**	Expressive aphasia	neuropsychiatric-speech-language
**HP:0033848**	Receptive aphasia	neuropsychiatric-speech-language
**HP:0001350**	Slurred speech	neuropsychiatric-speech-language
**HP:0001618**	Dysphonia	HEENT-ENT
**HP:0001742**	Nasal congestion	HEENT-ENT
**HP:0033050**	Pharyngalgia	HEENT-ENT
**HP:0012384**	Rhinitis	HEENT-ENT
**HP:0010524**	Agnosia	neuropsychiatric-cognitive-dysfunction
**HP:0002067**	Bradykinesia	neuropsychiatric-cognitive-dysfunction
**HP:0031843**	Bradyphrenia	neuropsychiatric-cognitive-dysfunction
**HP:0100543**	Cognitive impairment	neuropsychiatric-cognitive-dysfunction
**HP:0001289**	Confusion	neuropsychiatric-cognitive-dysfunction
**HP:0031987**	Diminished ability to concentrate	neuropsychiatric-cognitive-dysfunction
**HP:0001298**	Encephalopathy	neuropsychiatric-cognitive-dysfunction
**HP:0033844**	Tachyphrenia	neuropsychiatric-cognitive-dysfunction
**HP:0001596**	Alopecia	skin-findings
**HP:0011971**	Dermatographic urticaria	skin-findings
**HP:0031284**	Flushing	skin-findings
**HP:0001808**	Fragile nails	skin-findings
**HP:0000975**	Hyperhidrosis	skin-findings
**HP:0000967**	Petechiae	skin-findings
**HP:0000989**	Pruritus	skin-findings
**HP:0033696**	Pseudo-chilblain	skin-findings
**HP:0040189**	Scaling skin	skin-findings
**HP:0000988**	Skin rash	skin-findings
**HP:0031983**	Abnormal pulmonary thoracic imaging finding	pulmonary-imaging
**HP:0100750**	Atelectasis	pulmonary-imaging
**HP:0002110**	Bronchiectasis	pulmonary-imaging
**HP:0025180**	Centrilobular ground-glass opacification on pulmonary HRCT	pulmonary-imaging
**HP:0033659**	Crazy-paving pattern	pulmonary-imaging
**HP:0030879**	Interlobular septal thickening	pulmonary-imaging
**HP:0032177**	Parenchymal consolidation	pulmonary-imaging
**HP:0031944**	Pleural thickening	pulmonary-imaging
**HP:0032446**	Pulmonary bulla	pulmonary-imaging

medRxiv preprint doi: https://doi.org/10.64898/2026.07.06.26357401; this version posted July 9, 2026. The copyright holder for this preprint (which was not certified by peer review) is the author/funder, who has granted medRxiv a license to display the preprint in perpetuity.
It is made available under a CC-BY 4.0 International license .

**HP:0002206**	Pulmonary fibrosis	pulmonary-imaging
**HP:0033711**	Pulmonary interstitial thickening	pulmonary-imaging
**HP:0025390**	Reticular pattern on pulmonary HRCT	pulmonary-imaging
**HP:0033609**	Solid pulmonary nodule	pulmonary-imaging
**HP:0033610**	Subsolid pulmonary nodule	pulmonary-imaging
**HP:0033702**	Subpleural curvilinear line	pulmonary-imaging
**HP:0012213**	Decreased glomerular filtration rate	reproductive-genitourinary-endocrine-metabolism
**HP:0000819**	Diabetes mellitus	reproductive-genitourinary-endocrine-metabolism
**HP:0000969**	Edema	reproductive-genitourinary-endocrine-metabolism
**HP:0030014**	Female sexual dysfunction	reproductive-genitourinary-endocrine-metabolism
**HP:0001945**	Fever	reproductive-genitourinary-endocrine-metabolism
**HP:0002046**	Heat intolerance	reproductive-genitourinary-endocrine-metabolism
**HP:0002045**	Hypothermia	reproductive-genitourinary-endocrine-metabolism
**HP:0000858**	Irregular menstruation	reproductive-genitourinary-endocrine-metabolism
**HP:0011134**	Low-grade fever	reproductive-genitourinary-endocrine-metabolism
**HP:0040307**	Male sexual dysfunction	reproductive-genitourinary-endocrine-metabolism
**HP:0000132**	Menorrhagia	reproductive-genitourinary-endocrine-metabolism
**HP:0001733**	Pancreatitis	reproductive-genitourinary-endocrine-metabolism
**HP:0033840**	Postmenopausal bleeding	reproductive-genitourinary-endocrine-metabolism
**HP:0001954**	Recurrent fever	reproductive-genitourinary-endocrine-metabolism
**HP:0000083**	Renal insufficiency	reproductive-genitourinary-endocrine-metabolism
**HP:0005968**	Temperature instability	reproductive-genitourinary-endocrine-metabolism
**HP:0033839**	Testicular pain	reproductive-genitourinary-endocrine-metabolism
**HP:0000020**	Urinary incontinence	reproductive-genitourinary-endocrine-metabolism
**HP:0006536**	Airway obstruction	pulmonary-finding
**HP:0045051**	Decreased DLCO	pulmonary-finding
**HP:0033760**	Decreased maximal oxygen uptake	pulmonary-finding
**HP:0033773**	Decreased RV/TLC ratio	pulmonary-finding
**HP:0025179**	Ground-glass opacification	pulmonary-finding
**HP:0012418**	Hypoxemia	pulmonary-finding
**HP:0030874**	Oxygen desaturation on exertion	pulmonary-finding
**HP:0030877**	Reduced FEV1/FVC ratio	pulmonary-finding
**HP:0032342**	Reduced forced expiratory volume in one second	pulmonary-finding
**HP:0032341**	Reduced forced vital capacity	pulmonary-finding
**HP:0033845**	Sense of impending doom	neuropsychiatric-emotion-mood
**HP:0031589**	Suicidal ideation	neuropsychiatric-emotion-mood
**HP:0033705**	Tearfulness	neuropsychiatric-emotion-mood
**HP:0033689**	Anterograde memory impairment	neuropsychiatric-memory
**HP:0033688**	Long term memory impairment	neuropsychiatric-memory
**HP:0002354**	Memory impairment	neuropsychiatric-memory
**HP:0033691**	Procedural memory loss	neuropsychiatric-memory

medRxiv preprint doi: https://doi.org/10.64898/2026.07.06.26357401; this version posted July 9, 2026. The copyright holder for this preprint (which was not certified by peer review) is the author/funder, who has granted medRxiv a license to display the preprint in perpetuity.
It is made available under a CC-BY 4.0 International license .

**HP:0033687**	Short term memory impairment	neuropsychiatric-memory
**HP:0002829**	Arthralgia	General-pain
**HP:0033047**	Body ache	General-pain
**HP:0002653**	Bone pain	General-pain
**HP:0100749**	Chest pain	General-pain
**HP:0033746**	Intrascapular pain	General-pain
**HP:0009763**	Limb pain	General-pain
**HP:0003326**	Myalgia	General-pain
**HP:0033345**	Neuralgia	General-pain
**HP:0012531**	Pain	General-pain
**HP:0002020**	Gastroesophageal reflux	gi-findings
**HP:0002578**	Gastroparesis	gi-findings
**HP:0002592**	Gastric ulcer	gi-findings
**HP:0001397**	Hepatic steatosis	gi-findings
**HP:0002240**	Hepatomegaly	gi-findings
**HP:0012115**	Hepatitis	gi-findings
**HP:0004395**	Malnutrition	gi-findings
**HP:0033757**	Pancreatic steatosis	gi-findings
**HP:0001744**	Splenomegaly	gi-findings
**HP:0012053**	Decreased circulating calcifediol concentration	Lab
**HP:0003155**	Elevated circulating alkaline phosphatase concentration	Lab
**HP:0031964**	Elevated circulating alanine aminotransferase concentration	Lab
**HP:0031956**	Elevated circulating aspartate aminotransferase concentration	Lab
**HP:0003259**	Elevated circulating creatinine concentration	Lab
**HP:0003236**	Elevated circulating creatine kinase concentration	Lab
**HP:0011227**	Elevated circulating C-reactive protein concentration	Lab
**HP:0033106**	Elevated circulating D-dimer concentration	Lab
**HP:0033833**	Elevated circulating soluble CD25 concentration	Lab
**HP:0002925**	Elevated circulating thyroid-stimulating hormone concentration	Lab
**HP:0003565**	Elevated erythrocyte sedimentation rate	Lab
**HP:0030948**	Elevated gamma-glutamyltransferase level	Lab
**HP:0002901**	Hypocalcemia	Lab
**HP:0011900**	Hypofibrinogenemia	Lab
**HP:0003074**	Hyperglycemia	Lab
**HP:0001943**	Hypoglycemia	Lab
**HP:0002148**	Hypophosphatemia	Lab
**HP:0003281**	Increased circulating ferritin concentration	Lab
**HP:0030783**	Increased circulating interleukin 6	Lab
**HP:0025435**	Increased circulating lactate dehydrogenase concentration	Lab
**HP:0031185**	Increased circulating NT-proBNP concentration	Lab
**HP:0032308**	Increased circulating procalcitonin concentration	Lab

medRxiv preprint doi: https://doi.org/10.64898/2026.07.06.26357401; this version posted July 9, 2026. The copyright holder for this preprint (which was not certified by peer review) is the author/funder, who has granted medRxiv a license to display the preprint in perpetuity.
It is made available under a CC-BY 4.0 International license .

**HP:0001873**	Thrombocytopenia	Lab
**HP:0001681**	Angina pectoris	cardiovascular-symptom
**HP:0001962**	Palpitations	cardiovascular-symptom
**HP:0001297**	Stroke	cardiovascular-symptom
**HP:0001279**	Syncope	cardiovascular-symptom
**HP:0000739**	Anxiety	neuropsychiatric-behavioral
**HP:0000741**	Apathy	neuropsychiatric-behavioral
**HP:0007018**	Attention deficit hyperactivity disorder	neuropsychiatric-behavioral
**HP:0008765**	Auditory hallucinations	neuropsychiatric-behavioral
**HP:0033630**	Brain fog	neuropsychiatric-behavioral
**HP:0000746**	Delusions	neuropsychiatric-behavioral
**HP:0000738**	Hallucinations	neuropsychiatric-behavioral
**HP:0033051**	Impaired executive functioning	neuropsychiatric-behavioral
**HP:0100710**	Impulsivity	neuropsychiatric-behavioral
**HP:0000737**	Irritability	neuropsychiatric-behavioral
**HP:0025269**	Panic attack	neuropsychiatric-behavioral
**HP:0002183**	Phonophobia	neuropsychiatric-behavioral
**HP:0001959**	Polydipsia	neuropsychiatric-behavioral
**HP:0033676**	Posttraumatic stress symptom	neuropsychiatric-behavioral
**HP:0000736**	Short attention span	neuropsychiatric-behavioral
**HP:0033694**	Tactile hallucination	neuropsychiatric-behavioral
**HP:0002367**	Visual hallucinations	neuropsychiatric-behavioral
**HP:0012735**	Cough	pulmonary-symptom
**HP:0002094**	Dyspnea	pulmonary-symptom
**HP:0033750**	Reduced functional residual capacity	pulmonary-finding
**HP:0033753**	Reduced residual volume	pulmonary-finding
**HP:0033169**	Reduced total lung capacity	pulmonary-finding
**HP:0002091**	Restrictive ventilatory defect	pulmonary-finding
**HP:0002102**	Pleuritis	pulmonary-finding
**HP:0002204**	Pulmonary embolism	pulmonary-finding
**HP:0100845**	Anaphylactic shock	immunology-autoimmunity
**HP:0003493**	Antinuclear antibody positivity	immunology-autoimmunity
**HP:0025379**	Anti-thyroid peroxidase antibody positivity	immunology-autoimmunity
**HP:0032069**	Anti-thyroglobulin antibody positivity	immunology-autoimmunity
**HP:0002716**	Lymphadenopathy	immunology-autoimmunity
**HP:0001888**	Lymphopenia	immunology-autoimmunity
**HP:0002315**	Headache	neuropsychiatric-headache
**HP:0002076**	Migraine	neuropsychiatric-headache
**HP:0030766**	Ear pain	HEENT-ear
**HP:0000365**	Hearing impairment	HEENT-ear
**HP:0010780**	Hyperacusis	HEENT-ear

medRxiv preprint doi: https://doi.org/10.64898/2026.07.06.26357401; this version posted July 9, 2026. The copyright holder for this preprint (which was not certified by peer review) is the author/funder, who has granted medRxiv a license to display the preprint in perpetuity.
It is made available under a CC-BY 4.0 International license .

**HP:0008629**	Pulsatile tinnitus	HEENT-ear
**HP:0000360**	Tinnitus	HEENT-ear
**HP:0002321**	Vertigo	HEENT-ear
**HP:0041051**	Ageusia	neuropsychiatric-smell-taste
**HP:0000458**	Anosmia	neuropsychiatric-smell-taste
**HP:0000224**	Hypogeusia	neuropsychiatric-smell-taste
**HP:0004409**	Hyposmia	neuropsychiatric-smell-taste
**HP:0031249**	Parageusia	neuropsychiatric-smell-taste
**HP:0033847**	Phantageusia	neuropsychiatric-smell-taste
**HP:0033693**	Phantosmia	neuropsychiatric-smell-taste
**HP:0001369**	Arthritis	General-symptom
**HP:0025406**	Asthenia	General-symptom
**HP:0031352**	Chest tightness	General-symptom
**HP:0025143**	Chills	General-symptom
**HP:0033850**	Coldness	General-symptom
**HP:0002355**	Difficulty walking	General-symptom
**HP:0033665**	Diminished health-related quality of life	General-symptom
**HP:0033667**	Diminished mental health	General-symptom
**HP:0033666**	Diminished physical functioning	General-symptom
**HP:0003546**	Exercise intolerance	General-symptom
**HP:0012378**	Fatigue	General-symptom
**HP:0033675**	Frailty	General-symptom
**HP:0031059**	Impaired ability to bathe oneself	General-symptom
**HP:0031060**	Impaired ability to dress oneself	General-symptom
**HP:0031058**	Impairment of activities of daily living	General-symptom
**HP:0033834**	Malaise	General-symptom
**HP:0030166**	Night sweats	General-symptom
**HP:0033695**	Occupational disability	General-symptom
**HP:0030973**	Postexertional malaise	General-symptom
**HP:0025144**	Shivering	General-symptom
**HP:0025258**	Stiff neck	General-symptom
**HP:0001824**	Weight loss	General-symptom
**HP:0000217**	Xerostomia	General-symptom
**HP:0001662**	Bradycardia	cardiovascular-finding
**HP:4000006**	Elevated myocardial native T1	cardiovascular-finding
**HP:4000003**	Elevated myocardial native T2	cardiovascular-finding
**HP:0000822**	Hypertension	cardiovascular-finding
**HP:0002615**	Hypotension	cardiovascular-finding
**HP:0410174**	Increased circulating troponin T concentration	cardiovascular-finding
**HP:0410173**	Increased circulating troponin I concentration	cardiovascular-finding
**HP:0031862**	Increased heart rate variability	cardiovascular-finding

medRxiv preprint doi: https://doi.org/10.64898/2026.07.06.26357401; this version posted July 9, 2026. The copyright holder for this preprint (which was not certified by peer review) is the author/funder, who has granted medRxiv a license to display the preprint in perpetuity.
It is made available under a CC-BY 4.0 International license .

**HP:0033755**	Increased left ventricular end-diastolic volume	cardiovascular-finding
**HP:4000004**	Myocardial late gadolinium enhancement	cardiovascular-finding
**HP:0012819**	Myocarditis	cardiovascular-finding
**HP:0001698**	Pericardial effusion	cardiovascular-finding
**HP:4000005**	Pericardial late gadolinium enhancement	cardiovascular-finding
**HP:0012664**	Reduced ejection fraction	cardiovascular-finding
**HP:0001649**	Tachycardia	cardiovascular-finding
**HP:0004936**	Venous thrombosis	cardiovascular-finding
**HP:0000718**	Aggressive behavior	neuropsychiatric-emotion-mood
**HP:0000716**	Depression	neuropsychiatric-emotion-mood
**HP:0033838**	Dysphoria	neuropsychiatric-emotion-mood
**HP:0000712**	Emotional lability	neuropsychiatric-emotion-mood
**HP:0031844**	Euphoria	neuropsychiatric-emotion-mood
**HP:0100754**	Mania	neuropsychiatric-emotion-mood
**HP:0002875**	Exertional dyspnea	pulmonary-symptom
**HP:0002105**	Hemoptysis	pulmonary-symptom
**HP:0033709**	Increased sputum production	pulmonary-symptom
**HP:0031246**	Nonproductive cough	pulmonary-symptom
**HP:0033771**	Pleuritic chest pain	pulmonary-symptom
**HP:0031245**	Productive cough	pulmonary-symptom
**HP:0033710**	Rest dyspnea	pulmonary-symptom
**HP:0031417**	Rhinorrhea	pulmonary-symptom
**HP:0030831**	Rhonchi	pulmonary-symptom
**HP:0025095**	Sneeze	pulmonary-symptom
**HP:0002789**	Tachypnea	pulmonary-symptom
**HP:0030828**	Wheezing	pulmonary-symptom
**HP:0033747**	Abnormal exteroceptive sensation	neuropsychiatric-finding
**HP:0031826**	Abnormal reflex	neuropsychiatric-finding
**HP:0100022**	Abnormality of movement	neuropsychiatric-finding
**HP:0001251**	Ataxia	neuropsychiatric-finding
**HP:0003487**	Babinski sign	neuropsychiatric-finding
**HP:0001260**	Dysarthria	neuropsychiatric-finding
**HP:0001310**	Dysmetria	neuropsychiatric-finding
**HP:0002015**	Dysphagia	neuropsychiatric-finding
**HP:0001332**	Dystonia	neuropsychiatric-finding
**HP:0007209**	Facial paralysis	neuropsychiatric-finding
**HP:0000743**	Frontal release signs	neuropsychiatric-finding
**HP:0001288**	Gait disturbance	neuropsychiatric-finding
**HP:0030237**	Hand muscle weakness	neuropsychiatric-finding
**HP:0100963**	Hyperesthesia	neuropsychiatric-finding
**HP:0002487**	Hyperkinetic movements	neuropsychiatric-finding

medRxiv preprint doi: https://doi.org/10.64898/2026.07.06.26357401; this version posted July 9, 2026. The copyright holder for this preprint (which was not certified by peer review) is the author/funder, who has granted medRxiv a license to display the preprint in perpetuity.
It is made available under a CC-BY 4.0 International license .

**HP:0033748**	Hypoesthesia	neuropsychiatric-finding
**HP:0001252**	Hypotonia	neuropsychiatric-finding
**HP:0003394**	Muscle spasm	neuropsychiatric-finding
**HP:0001324**	Muscle weakness	neuropsychiatric-finding
**HP:0001278**	Orthostatic hypotension	neuropsychiatric-finding
**HP:0003401**	Paresthesia	neuropsychiatric-finding
**HP:0001300**	Parkinsonism	neuropsychiatric-finding
**HP:0001271**	Polyneuropathy	neuropsychiatric-finding
**HP:0002063**	Rigidity	neuropsychiatric-finding
**HP:0001250**	Seizure	neuropsychiatric-finding
**HP:0003202**	Skeletal muscle atrophy	neuropsychiatric-finding
**HP:0003474**	Somatic sensory dysfunction	neuropsychiatric-finding
**HP:0001257**	Spasticity	neuropsychiatric-finding
**HP:0001337**	Tremor	neuropsychiatric-finding
**HP:0012799**	Unilateral facial palsy	neuropsychiatric-finding
**HP:0100785**	Insomnia	neuropsychiatric-sleep
**HP:0031355**	Maintenance insomnia	neuropsychiatric-sleep
**HP:0012452**	Restless legs	neuropsychiatric-sleep
**HP:0010535**	Sleep apnea	neuropsychiatric-sleep
**HP:0002360**	Sleep disturbance	neuropsychiatric-sleep
**HP:0031354**	Sleep onset insomnia	neuropsychiatric-sleep
**HP:0031356**	Terminal insomnia	neuropsychiatric-sleep
**HP:0002027**	Abdominal pain	gi-symptoms
**HP:0011458**	Abdominal symptom	gi-symptoms
**HP:0002039**	Anorexia	gi-symptoms
**HP:0002607**	Bowel incontinence	gi-symptoms
**HP:0002019**	Constipation	gi-symptoms
**HP:0002014**	Diarrhea	gi-symptoms
**HP:0033842**	Early satiety	gi-symptoms
**HP:0002018**	Nausea	gi-symptoms
**HP:0002013**	Vomiting	gi-symptoms
**HP:0004396**	Poor appetite	gi-symptoms
**HP:0000618**	Blindness	HEENT-eye
**HP:0000622**	Blurred vision	HEENT-eye
**HP:0000509**	Conjunctivitis	HEENT-eye
**HP:0000651**	Diplopia	HEENT-eye
**HP:0000640**	Gaze-evoked nystagmus	HEENT-eye
**HP:0001097**	Keratoconjunctivitis sicca	HEENT-eye
**HP:0200026**	Ocular pain	HEENT-eye
**HP:0033841**	Ocular pruritus	HEENT-eye
**HP:0007994**	Peripheral visual field loss	HEENT-eye

medRxiv preprint doi: https://doi.org/10.64898/2026.07.06.26357401; this version posted July 9, 2026. The copyright holder for this preprint (which was not certified by peer review) is the author/funder, who has granted medRxiv a license to display the preprint in perpetuity.
It is made available under a CC-BY 4.0 International license .

**HP:0000613**	Photophobia	HEENT-eye
**HP:0025337**	Red eye	HEENT-eye
**HP:0000572**	Visual loss	HEENT-eye
**HP:0100832**	Vitreous floaters	HEENT-eye
